# Trends in purchasing cross‐border, illicit and home‐brewed alcohol: A population study in Great Britain, 2020–2023

**DOI:** 10.1111/dar.13838

**Published:** 2024-03-21

**Authors:** Sarah E. Jackson, Melissa Oldham, Colin Angus, John Holmes, Jamie Brown

**Affiliations:** ^1^ Department of Behavioural Science and Health University College London London UK; ^2^ SPECTRUM Consortium Edinburgh UK; ^3^ Division of Population Health University of Sheffield Sheffield UK

**Keywords:** alcohol, cross‐border, duty free, home‐brew, illicit

## Abstract

**Introduction:**

The last 3 years have seen substantial changes in Great Britain (GB) including the COVID‐19 pandemic, cost‐of‐living crisis and policy changes such as minimum unit pricing. We examined changes in purchasing cross‐border, illicit and home‐brewed alcohol among risky drinkers over this period.

**Methods:**

Data were used from 22,086 adult (≥18 years) increasing/higher‐risk drinkers (AUDIT‐C ≥5) participating in a monthly cross‐sectional survey between October 2020 and August 2023. We estimated time trends in the proportion reporting obtaining alcohol from: (i) cross‐border (any/within‐GB/international); (ii) illicit; and (iii) home‐brewed sources in the past 6 months.

**Results:**

Between October 2020 and August 2023, the proportion reporting cross‐border alcohol purchases increased (from 8.5% to 12.5% overall; prevalence ratio [PR] = 1.47 [95% CI 1.17–1.86]). This was largely driven by an increase in cross‐border purchases abroad (PR = 1.52 [1.13–2.05]), with a smaller, uncertain increase in cross‐border purchases within GB (PR = 1.37 [0.96–1.95]). The prevalence of cross‐border alcohol purchasing was higher in Wales (13.8% [12.3–15.4%]) and Scotland (6.1% [5.4–6.8%]) than England (3.6% [3.3–3.9%]). There was little change in illicit alcohol purchasing in England or Wales (4.1% [3.7–4.4%]; 4.2% [3.2–5.1%]), but in Scotland it fell from 5.7% to 2.4% (PR = 0.42 [0.19–0.81]). Home‐brewed alcohol was rare (GB: 3.1% [2.9–3.4]) and stable.

**Discussion and Conclusions:**

The proportion of increasing/higher‐risk drinkers in GB purchasing cross‐border alcohol increased between October 2020 and August 2023, due to an increase in people buying alcohol abroad. Cross‐border alcohol purchases within GB were more commonly reported in Wales and Scotland. The small proportion purchasing illicit alcohol did not change substantially in England or Wales, but fell by half in Scotland.

## 1 INTRODUCTION

Increasing the price of alcohol, through alcohol taxation or other pricing policies, has long been recognised as an effective and cost‐effective way to reduce harmful alcohol consumption and harm [1, 2] and reduce health inequalities [3]. Alcohol tax avoidance and tax evasion strategies, or other approaches to avoid paying the higher prices resulting from these policies, undermines their effectiveness. Understanding how prevalent use of these strategies is and how this is changing over time is important for informing policy.

Tax avoidance strategies include purchasing alcohol legally from lower‐tax or price jurisdictions across jurisdictional borders (e.g., between states or countries), or duty‐free while travelling between countries (‘cross‐border purchases’) [4, 5]. Across countries, there is wide variation in both the scale and the structure of alcohol taxation [6]. Previous work in this area has focused particularly on Northern Europe and the impact on cross‐border purchasing of large tax changes or changes in importation rules as countries joined the European Union (EU) or other trading areas. These studies generally show high levels of cross‐border trade, although changes in such trade do not always follow policy shifts [7, 8, 9]. The United Kingdom (UK) levies high duty rates compared with many other countries in Europe: in 2018, the effective duty rate per unit of alcohol (1 UK unit = 10 mL/8 g ethanol) in the UK was at least six times higher than the lowest‐duty country across different beverage types (beer, wine and spirits) [10]. Although alcohol duties do not differ across the countries of the UK, there are significant differences in other aspects of alcohol policy. Most significantly, the Scottish Government introduced minimum unit pricing (MUP) in May 2018, which sets a floor price of £0.50 per unit of alcohol below which retailers cannot sell alcohol drinks to consumers. The Welsh Government introduced MUP at the same rate in March 2020. When MUP was introduced in Scotland there was some concern that cross‐border purchasing could significantly undermine the effectiveness of MUP, but several studies [11, 12, 13] have found only limited evidence of increased cross‐border purchasing, and then only in people living closest to the border. However, as yet, the initial evidence emerging from Wales is inconclusive [14] and the fact that many more people in Wales than in Scotland live within easy driving distance of England suggests there may be more reason to be concerned about the potential for cross‐border purchasing to significantly undermine the potential effectiveness of MUP.

Tax evasion strategies include deliberately obtaining alcohol from illegal sources where no tax is paid at all, such as smuggling or buying counterfeit alcohol (‘illicit purchases’) [15, 16]. Illicit alcohol lacks the regulatory and market oversight that legal alcohol products would have, increasing the risk of quality and safety issues and loss of revenue through taxation [15, 16].

In addition to these tax avoidance and tax evasion strategies, some people manufacture their alcohol at home and therefore do not pay alcohol duty. In the UK, home brewing is legal for personal, non‐commercial purposes but home distilling is not.

Several factors are likely to have affected the availability of, and people's motivation to use, low‐price or untaxed alcohol in recent years. First, the introduction of MUP in Scotland and Wales has increased the price of alcohol per gram by around 8% on average, and by much larger amounts for the cheapest products [17, 18]. Those living near borders may travel to England, where MUP is not in place, to take advantage of cheaper prices. Second, as a result of Brexit, people arriving in the UK from countries within the EU after January 2021 could no longer bring back unlimited quantities of locally paid duty/tax alcohol intended for personal consumption. Instead, people arriving to the UK from the EU and the rest of the world have a duty‐free allowance, with purchases exceeding this quantity attracting UK duty [19]. This change introduced a duty‐free allowance for the EU while increasing the allowance from outside the EU (42 L of beer, 18 L of still wine, and either 4 L of spirits or similar or 9 L of sparkling wine or similar). Third, the COVID‐19 pandemic (from March 2020) restricted social interaction and national and international travel, which may have reduced people's access to cheap alcohol, changed the balance of their purchasing of alcohol in shops relative to bars, and affected their alcohol consumption and motivations to drink for other reasons unrelated to price [20, 21]. Finally, the pandemic and, more recently, the ongoing cost‐of‐living crisis (since late 2021) have exposed many people to financial hardship as a result of loss of earnings [22] and the cost of everyday essentials like groceries, household and energy bills rising faster than average household incomes [23, 24]. This may have increased motivation to purchase alcohol from low price or untaxed sources to reduce the cost of drinking [25], particularly among less advantaged socioeconomic groups and those buying larger amounts of alcohol [26, 27]. On the other hand, it may have limited people's financial means to travel—either to take international holidays, or access to everyday travel (e.g., owning a car), particularly among less advantaged socioeconomic groups.

The Alcohol Toolkit Study (a representative, monthly cross‐sectional survey) has been collecting data on where increasing‐ and higher‐risk drinkers in Great Britain (GB) purchase their alcohol since October 2020. This study aimed to use these data to address the following research questions:

1. What proportion of increasing‐ and higher‐risk drinkers (defined as a score ≥5 on the AUDIT‐C [28]) report purchasing cross‐border, illicit and home‐brewed alcohol, and how does this vary between countries within GB (England, Scotland and Wales)?
2. How have cross‐border (within GB and abroad), illicit and home‐brewed alcohol purchasing changed between 2020 and 2023?
3. Have trends varied between countries within GB?
4. Have trends (in GB overall, or in England, Scotland or Wales) varied by socioeconomic position?

## 2 METHOD

### 2.1 Pre‐registration

The study protocol and analysis plan were pre‐registered on Open Science Framework (https://osf.io/4gwbu/). We made one amendment: we had specified that we would model time trends using restricted cubic splines with three knots. However, visual inspection of the modelled trends against unmodelled data points indicated a poor fit, so we increased the number of knots to four to allow greater flexibility.

### 2.2 Design

Data were drawn from the Alcohol Toolkit Study, an ongoing monthly cross‐sectional survey of a nationally representative sample of adults in GB [29, 30]. The study uses a hybrid of random probability and quota sampling to select a new sample of approximately 2450 adults each month (1700 in England, 450 in Scotland and 300 in Wales). Data are collected monthly through computer‐assisted telephone interviews. Survey weights are derived using raking to match each monthly sample to the population in GB on the dimensions of age, social grade, region, housing tenure, ethnicity and working status within sex [31]. The demographic profile of the population in GB is determined each month by combining data from the 2021 UK Census, the Office for National Statistics mid‐year estimates and the annual National Readership Survey [32]. This enables data to be pooled across survey waves, while allowing each monthly sample to be weighted to match the population at the time the data were collected. Full details of the sampling procedure are provided elsewhere [29, 30].

For the present study, we used data collected from participants in the period from October 2020 (the first wave in which data collection was expanded to included Scotland and Wales) to August 2023 (the most recent data available at the time of analysis) who were aged ≥18 years and reported drinking at increasing‐ or higher‐risk levels (defined as a score ≥5 on the AUDIT‐C [28]). Source of alcohol purchases was not assessed among participants in England in certain waves during this period (May, July, September, November and December 2022 and July 2023) so we only included those surveyed in Scotland and Wales in these months.

### 2.3 Measures

#### 2.3.1 Sources of alcohol purchases

Where increasing‐ and higher‐risk drinkers purchase their alcohol was assessed with the question: ‘In the last 6 months, have you bought any alcohol for your consumption from any of the following?’. Participants could select multiple responses from a list of 15 options (see protocol for full list) including various retail outlets and informal sources.

Cross‐border purchasing was coded 1 for those who reported buying alcohol from abroad and bringing it back with them or buying from another country within GB, else it was coded 0. Duty‐free sources within GB were not specified as a response option and some respondents may have included these in their definition of cross‐border sources. We also analysed cross‐border purchasing within‐GB and abroad as separate outcomes.

Purchase from illicit sources was coded 1 for those who reported buying alcohol under the counter (from newsagent, off‐licence, corner shop, or pub), from people who sell cheaply on the street (or car parks, etc.), from people in the local area who are a ready supply of cheap alcohol, or cheap from friends, else it was coded 0.

Home brewing was coded 1 for those who reported buying home‐brewed alcohol, else it was coded 0.

#### 2.3.2 Country within Great Britain

Participants' country of residence was categorised as England, Wales or Scotland.

#### 2.3.3 Occupational social grade

Social grade was categorised based on National Readership Survey classifications [33] as ABC1, which includes managerial, professional and upper supervisory occupations and C2DE, which includes manual routine, semi‐routine, lower supervisory and long‐term unemployed.

### 2.4 Statistical analysis

Data were analysed in R v.4.2.1. All analyses used weighted data.

#### 2.4.1 RQ1: Prevalence

Using data aggregated across the study period, we calculated the weighted proportion and 95% confidence interval (CI) of increasing‐ and higher‐risk drinkers reporting purchasing: (i) cross‐border alcohol (any cross‐border; within‐GB; and abroad); (ii) illicit alcohol; and (iii) home brewed alcohol in the past 6 months. We reported estimates for the entire GB sample and stratified by nation and social grade.

#### 2.4.2 RQ2‐4: Time trends

We used logistic regression (using the svyglm package) to estimate monthly time trends in the proportion of increasing‐ and higher‐risk drinkers purchasing: (i) cross‐border alcohol (any cross‐border; within‐GB; and abroad); (ii) illicit alcohol; and (iii) home brewed alcohol in the past 6 months.

For the overall analysis (RQ2), models only included time (survey month) as a predictor. Survey month was modelled using restricted cubic splines with four knots, to allow relationships with time to be flexible and non‐linear, while avoiding categorisation.

For the country‐specific analyses (RQ3), models included time, country and their interaction as predictors—thus allowing for time trends to differ across countries.

For the social grade‐specific analyses (RQ4), we ran four models for each outcome (for GB overall, and separately for England, Scotland and Wales). Models included time, social grade, and their interaction as predictors—thus allowing for time trends (overall and within each country) to differ across social grades.

We used predicted estimates from our models to plot the prevalence of each outcome over the study period (overall and by country and social grade), alongside unmodelled (weighted) monthly data points. Prevalence ratios (PR) for changes in prevalence across the whole time‐series (calculated as the predicted estimate of prevalence in August 2023 divided by the predicted estimate of prevalence in October 2020) are presented, alongside 95% CIs calculated using bootstrapping.

Following peer review, we updated the analysis including data up to October 2023 so that the first and last waves of data included were collected in the same calendar month.

## 3 RESULTS

Of 79,352 participants surveyed in eligible waves, 25,225 (31.8%) reported drinking at increasing or higher‐risk levels. We excluded 3139 participants in England surveyed in waves that did not assess source of alcohol purchasing (May, July, September, November and December 2022 and July 2023). There were 1353 participants surveyed in Wales and Scotland across these six waves; they were excluded from whole‐GB analyses but retained for within‐country analyses. There were no missing data on country of residence or social grade. This left a final sample for analysis of 22,086 increasing‐ and higher‐risk drinkers (weighted descriptive statistics: mean [SD] age = 46.1 [16.8] years, 38.0% women, 38.5% social grades C2DE; see Table S1 for a comparison with all participants surveyed in eligible waves), of whom 20,733 contributed data to whole‐GB analyses.

### 3.1 Overall estimates of prevalence

Across the study period, 9.1% of increasing‐ and higher‐risk drinkers reported having purchased cross‐border alcohol in the past 6 months (4.3% within GB and 5.6% abroad^1^), 4.1% reported having purchased illicit alcohol, and 3.1% home brewed alcohol (Table 1).

**TABLE 1:** Unadjusted weighted prevalence of purchasing cross‐border, illicit and home‐brewed alcohol among increasing‐ and higher‐risk drinkers in Great Britain: data aggregated across the study period (October 2020–August 2023).

	Prevalence, % [95% CI]			
	Great Britain	England	Wales	Scotland
All increasing‐ and higher‐risk drinkers				
Unweighted *N*	20,733	14,547	2402	5137
Cross‐border alcohol^a^	9.1 [8.7–9.5]	8.6 [8.1–9.1]	17.2 [15.5–18.9]	10.9 [10.0–11.8]
Within GB	4.3 [4.0–4.6]	3.6 [3.3–3.9]	13.8 [12.3–15.4]	6.1 [5.4–6.8]
Abroad	5.6 [5.3–6.0]	5.7 [5.3–6.1]	5.9 [4.8–6.9]	6.0 [5.3–6.7]
Illicit alcohol^a^	4.1 [3.8–4.4]	4.1 [3.7–4.4]	4.2 [3.2–5.1]	3.9 [3.3–4.5]
Home‐brewed alcohol^a^	3.1 [2.9–3.4]	3.1 [2.8–3.4]	3.4 [2.6–4.1]	2.9 [2.3–3.4]
Social grades ABC1 (more advantaged)				
Unweighted *N*	14,765	10,476	1643	3600
Cross‐border alcohol^a^	10.1 [9.5–10.6]	9.6 [9.0–10.2]	19.2 [17.2–21.3]	11.2 [10.2–12.3]
Within GB	4.6 [4.3–5.0]	3.9 [3.5–4.3]	15.4 [13.5–17.4]	6.5 [5.7–7.4]
Abroad	6.4 [6.0–6.8]	6.5 [6.0–7.0]	7.1 [5.7–8.4]	5.9 [5.1–6.7]
Illicit alcohol^a^	3.8 [3.5–4.2]	3.8 [3.4–4.2]	4.1 [3.0–5.3]	3.6 [2.9–4.3]
Home‐brewed alcohol^a^	3.3 [3.0–3.6]	3.4 [3.0–3.8]	4.0 [2.9–5.0]	2.3 [1.8–2.8]
Social grades C2DE (less advantaged)				
Unweighted *N*	5968	4071	759	1537
Cross‐border alcohol^a^	7.5 [6.8–8.3]	6.9 [6.1–7.8]	14.4 [11.6–17.1]	10.4 [8.7–12.0]
Within GB	3.8 [3.2–4.3]	3.1 [2.5–3.7]	11.6 [9.1–14.1]	5.5 [4.3–6.7]
Abroad	4.4 [3.8–5.0]	4.3 [3.7–5.0]	4.2 [2.6–5.8]	6.1 [4.8–7.3]
Illicit alcohol^a^	4.5 [3.9–5.1]	4.5 [3.8–5.2]	4.2 [2.6–5.8]	4.4 [3.2–5.5]
Home‐brewed alcohol^a^	2.8 [2.4–3.3]	2.7 [2.2–3.3]	2.5 [1.3–3.6]	3.7 [2.6–4.7]

Abbreviations: CI, confidence interval; GB, Great Britain.

^a^ In the past 6 months.

The prevalence of cross‐border alcohol purchasing—specifically, cross‐border purchasing within GB—was highest in Wales and lowest in England (Table 1). The proportions who reported buying cross‐border alcohol abroad, illicit alcohol and home brewed alcohol were similar across countries (Table 1).

The prevalence of cross‐border alcohol purchasing—both abroad and, to some extent, within GB—was higher among those from more advantaged social grades (Table 1). The proportions who reported buying illicit and home brewed alcohol were similar across social grades (Table 1).

### 3.2 Time trends

**FIGURE 1:**
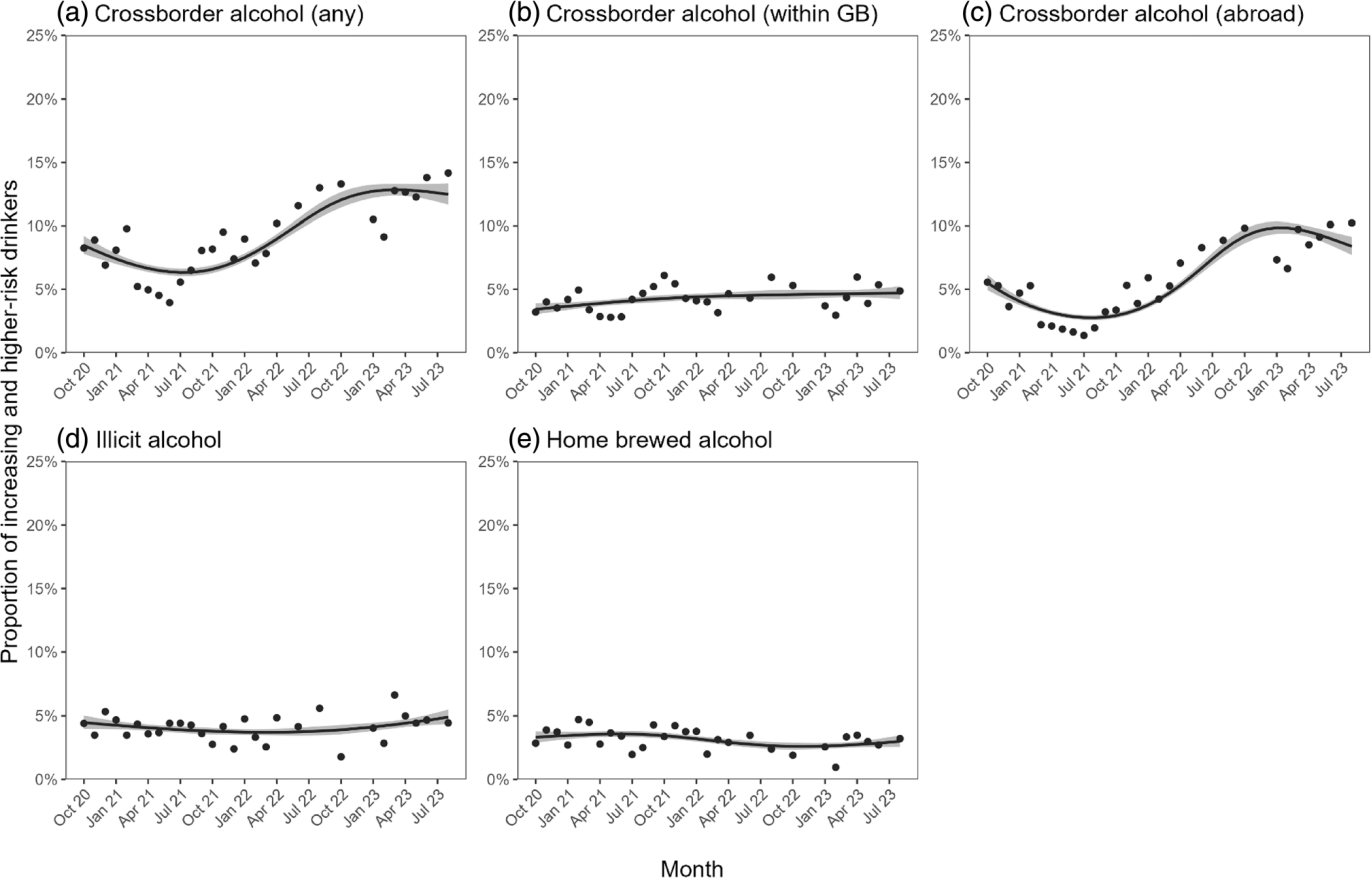
Trends in the proportion of increasing‐ and higher‐risk drinkers in Great Britain (*n* = 20,733) who reported purchasing cross‐border, illicit and home‐brewed alcohol, October 2020 to August 2023. Lines represent modelled weighted prevalence by monthly survey wave, modelled non‐linearly using restricted cubic splines (four knots). Shaded bands represent standard errors. Points represent observed weighted prevalence by month. Corresponding figures based on models that included data up to October 2023 are shown in Figure S1.

From October 2020 to August 2023, the proportion of participants reporting having purchased cross‐border alcohol in the past 6 months increased significantly (PR = 1.47 [95% CI 1.17–1.86]; Table 2). The change over time was non‐linear (Figure 1a), falling from 8.5% to 6.3% between October 2020 and July 2021, increasing to a peak of 12.8% in March 2023 and then falling slightly to 12.5% by August 2023.

**TABLE 2:** Changes in purchasing cross‐border, illicit and home‐brewed alcohol among increasing‐ and higher‐risk drinkers in Great Britain between October 2020 and August 2023.

	Cross‐border alcohol			Cross‐border alcohol within GB			Cross‐border alcohol abroad		
	% [95% CI]		Prevalence ratio Oct 20–Aug 23 [95% CI]	% [95% CI]		Prevalence ratio Oct 20–Aug 23 [95% CI]	% [95% CI]		Prevalence ratio Oct 20–Aug 23 [95% CI]
	October 2020^a^	August 2023^a^		October 2020^a^	August 2023^a^		October 2020^a^	August 2023^a^	
All increasing‐ and higher‐risk drinkers	8.5 [7.2–10.0]	12.5 [10.9–14.2]	1.47 [1.17–1.86]	3.4 [2.7–4.4]	4.7 [3.8–5.8]	1.37 [0.96–1.95]	5.5 [4.4–6.8]	8.4 [7.1–9.9]	1.52 [1.13–2.05]
Country									
England	7.7 [6.4–9.3]	11.9 [10.1–13.9]	1.54 [1.20–2.02]	2.7 [1.9–3.7]	4.0 [3.0–5.3]	1.49 [0.97–2.33]	5.4 [4.2–6.8]	8.5 [7.0–10.2]	1.58 [1.17–2.22]
Wales	17.7 [12.4–24.6]	19.2 [14.3–25.3]	1.08 [0.71–1.79]	14.9 [10.2–21.2]	13.9 [9.7–19.6]	0.93 [0.55–1.64]	4.3 [1.8–10.0]	7.2 [4.3–11.8]	1.67 [0.70–7.01]
Scotland	8.6 [6.1–12.0]	14.8 [11.7–18.5]	1.72 [1.13–2.78]	4.6 [2.9–7.1]	7.1 [5.0–9.9]	1.54 [0.85–2.84]	4.7 [2.8–7.9]	9.2 [6.8–12.4]	1.95 [1.11–4.07]
Social grade									
ABC1 (more advantaged)	9.6 [7.9–11.5]	14.6 [12.7–16.8]	1.54 [1.22–2.00]	3.8 [2.9–5.0]	5.4 [4.2–6.8]	1.41 [0.96–2.12]	6.6 [5.2–8.4]	10.1 [8.4–12.0]	1.52 [1.14–2.16]
C2DE (less advantaged)	6.8 [4.9–9.4]	9.1 [6.9–11.9]	1.29 [0.85–2.06]	2.9 [1.8–4.6]	3.7 [2.4–5.7]	1.29 [0.65–2.55]	3.9 [2.5–6.1]	5.9 [4.1–8.4]	1.44 [0.80–2.81]

	Illicit alcohol			Home‐brewed alcohol		
	% [95% CI]		Prevalence ratio Oct 20–Aug 23 [95% CI]	% [95% CI]		Prevalence ratio Oct 20–Aug 23 [95% CI]
	October 2020^a^	August 2023^a^		October 2020^a^	August 2023^a^	
All increasing‐ and higher‐risk drinkers	4.5 [3.6–5.6]	4.9 [3.9–6.1]	1.10 [0.78–1.54]	3.3 [2.2–4.0]	3.0 [2.2–4.0]	0.90 [0.59–1.38]
Country						
England	4.4 [3.4–5.7]	5.2 [4.0–6.7]	1.17 [0.81–1.70]	3.3 [2.4–4.3]	3.3 [2.4–4.6]	1.02 [0.65–1.61]
Wales	3.3 [1.5–7.1]	3.1 [1.2–7.7]	0.93 [0.20–3.48]	3.5 [1.7–6.8]	2.2 [1.0–4.7]	0.64 [0.19–1.95]
Scotland	5.7 [3.7–8.5]	2.4 [1.4–4.0]	0.42 [0.19–0.81]	3.4 [2.0–5.8]	1.4 [0.7–3.1]	0.43 [0.14–1.12]
Social grade						
ABC1 (more advantaged)	3.9 [2.9–5.2]	4.6 [3.5–6.1]	1.24 [0.82–1.90]	3.4 [2.5–4.5]	3.4 [2.5–4.6]	1.00 [0.64–1.59]
C2DE (less advantaged)	5.3 [3.7–7.6]	4.9 [3.3–7.3]	0.94 [0.53–1.69]	3.2 [2.0–5.2]	2.4 [1.2–4.5]	0.73 [0.29–1.70]

Abbreviation: CI, confidence interval.

^a^ Weighted prevalence from logistic regression models on all increasing‐ and higher‐risk drinkers in Great Britain and (for estimates by region and social grade) allowing an interaction between survey wave and the moderator of interest, modelled non‐linearly using restricted cubic splines. Estimates from models that included data up to October 2023 are provided in Table S2. Estimates by social grade within countries in Great Britain are provided in Table S3.

The rise in cross‐border alcohol purchasing was largely driven by cross‐border purchasing abroad (PR = 1.52 [95% CI 1.13–2.05]), with a smaller, uncertain increase in cross‐border purchasing within GB (PR = 1.37 [95% CI 0.96–1.95]; Table 2). The trend in cross‐border purchasing abroad mirrored the overall trend in cross‐border purchasing, falling from 5.5% to 2.8% between October 2020 and August 2021, increasing to 9.9% by January 2023, and then falling slightly to 8.4% by August 2023 (Figure 1c). Cross‐border purchasing within GB increased from 3.4% to 4.6% between October 2020 and November 2022, then remained relatively stable between 4.6% and 4.7% up to August 2023 (Figure 1b).

There was little change over time in the proportion reporting illicit (Figure 1d) or home‐brewed (Figure 1e) alcohol purchases (Table 2).

The pattern of results was not substantially different when we repeated the analyses including data up to October 2023 (Table S2 and Figure S1).

### 3.3 Time trends by country

**FIGURE 2:**
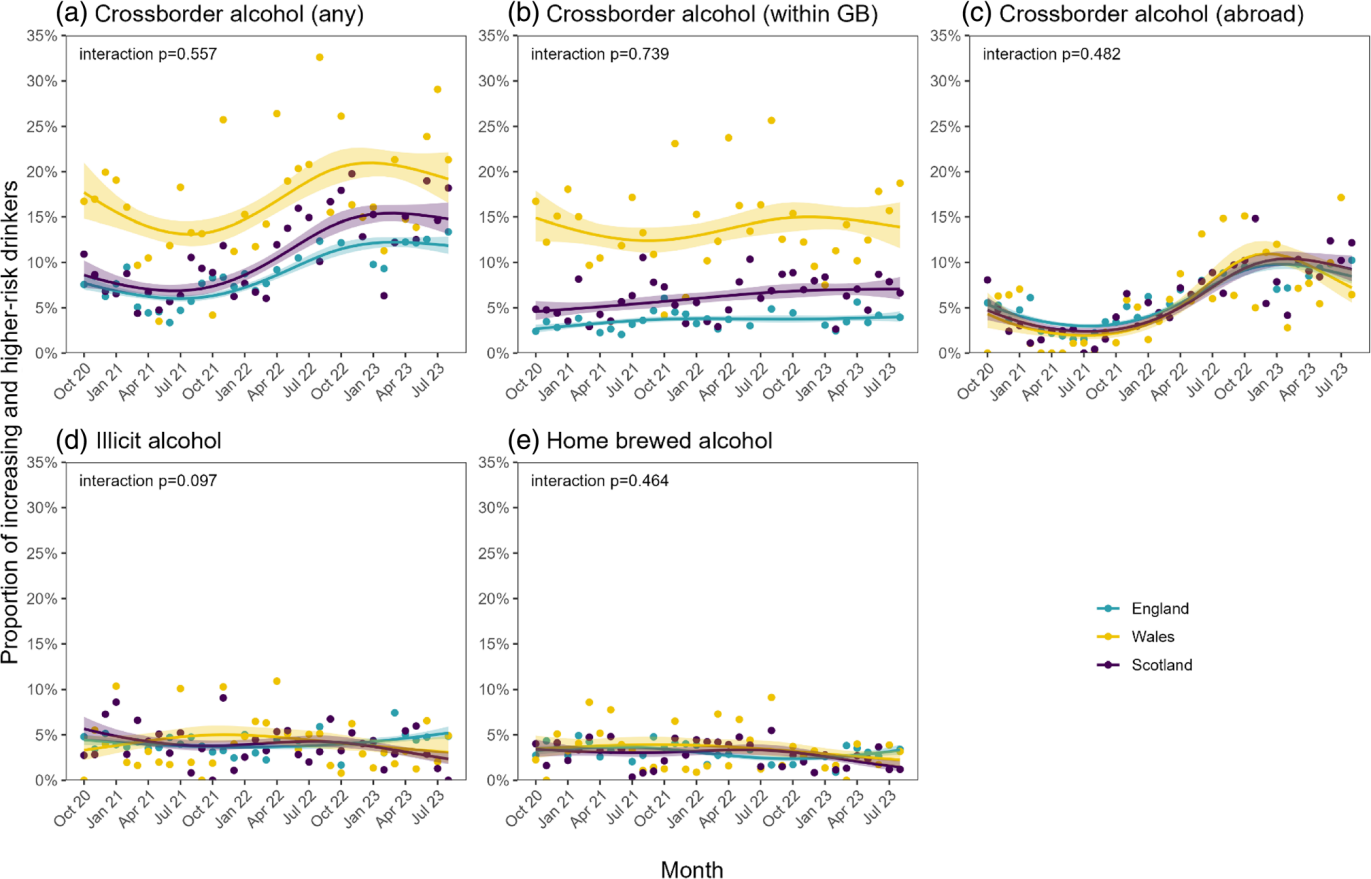
Trends in the proportion of increasing‐ and higher‐risk drinkers in England (*n* = 14,547), Wales (*n* = 2402) and Scotland (*n* = 5137) who reported purchasing cross‐border, illicit and home‐brewed alcohol, October 2020 to August 2023. Lines represent modelled weighted prevalence by monthly survey wave, modelled non‐linearly using restricted cubic splines (four knots). Shaded bands represent standard errors. Points represent observed weighted prevalence by month. Corresponding figures based on models that included data up to October 2023 are shown in Figure S2.

Monthly time trends in cross‐border, illicit and home‐brewed alcohol purchasing did not differ significantly between countries (Figure 2). However, there were some cross‐country differences in the overall change from the start to the end of the study period. We observed an increase in overall cross‐border alcohol purchasing in England (PR = 1.54 [95% CI 1.20–2.02]) and Scotland (PR = 1.72 [95% CI 1.13–2.78]), but not in Wales (PR = 1.08 [95% CI 0.71–1.79]; Table 2), driven by increases in cross‐border purchasing abroad for England and Scotland (PR = 1.58 [95% CI 1.17–2.22] and PR = 1.95 [95% CI 1.11–4.07]) and uncertain increases in cross‐border alcohol purchasing within GB in England (PR = 1.49 [95% CI 0.97–2.33]) and Scotland (PR = 1.54 [0.85–2.84]), but not in Wales for either (PR = 1.67 [95% CI 0.70–7.01]; PR = 0.93 [95% CI 0.55–1.64]; Table 2). Illicit alcohol purchasing fell by more than half in Scotland, from 5.7% to 2.4% (PR = 0.42 [95% CI 0.19–0.81]) but did not change significantly in England (PR = 1.17 [95% CI 0.81–1.70]) or Wales (PR = 0.93 [95% CI 0.20–3.48]; Table 2). There was also an uncertain fall in home‐brewed alcohol purchasing in Scotland (PR = 0.43 [95% CI 0.14–1.12]) but appeared more stable in England (PR = 1.02 [95% CI 0.65–1.61]) and Wales (PR = 0.64 [95% CI 0.19–1.95]; Table 2).

The pattern of results was not substantially different when we repeated the analyses including data up to October 2023 (Table S2 and Figure S2).

### 3.4 Time trends by social grade

Trends in cross‐border, illicit and home‐brewed alcohol purchasing did not differ significantly by social grade in GB (Table 2 and Figure S3), England (Figure S4), Wales (Figure S5) or Scotland (Figure S6).

## 4 DISCUSSION

Between October 2020 and August 2023, there was a non‐linear increase in the proportion of participants reporting cross‐border alcohol purchases. This largely reflected an increase in people buying alcohol abroad, with a smaller, uncertain increase in the proportion buying alcohol across borders within GB—these overall GB trends were mirrored in England and Scotland but not Wales. Across the period, the prevalence of cross‐border alcohol purchasing within GB was higher in Wales and Scotland than in England. There was little change over time in the proportion reporting illicit alcohol purchases in England or Wales, but in Scotland it fell by 50%. Home‐brewed alcohol purchasing remained rare and did not change significantly over time. Cross‐border purchasing was more prevalent among those from more vs. less advantaged social grades, but changes in cross‐border, illicit and home‐brewed alcohol purchasing over time were similar across social grades.

The curvilinear trend in cross‐border alcohol purchasing might be explained by changes in access resulting from the COVID‐19 pandemic. At various stages during the pandemic, there were restrictions on international travel both into and from the UK. In the UK, lockdown measures were first introduced in March 2020 and people were advised against ‘non‐essential’ international travel. From June 2020, all arrivals into the UK were required to self‐isolate (quarantine) [34]. In July 2020, although the government continued to advise against all but essential travel, restrictions were eased slightly and ‘travel corridors’ were introduced, which allowed people arriving from selected destinations to enter the UK without needing to self‐isolate [35]. Between 29 March and 16 May 2021, there was a ban on travel from England to destinations outside of the UK [36]. From 17 May 2021, non‐essential travel was allowed to resume, with testing and quarantine requirements on entry to the UK depending on the perceived level of risk in the country someone is entering from and their vaccination status [37]. All remaining UK travel restrictions were lifted in March 2022 [38]. The proportion of drinkers who reported purchasing alcohol abroad in the past 6 months declined substantially between October 2020 and June 2021, during a period in which restrictions on international travel were in place. This decline rebounded rapidly as people began travelling abroad again during the summer of 2021 [39], after the international travel ban was lifted, then fell slightly mirroring the seasonal pattern of overseas travel in 2021 (note that as our measure captures past‐6‐month purchases, there is a potential lag between time of purchasing and time of reporting). It is not clear from our data how far cross‐border alcohol purchasing abroad had already fallen by the start of the study period (October 2020), 8 months into the COVID‐19 pandemic, so changes from the start to the end of this period should be considered in the context of this almost certainly being a suppressed baseline [40]. There may also have been impacts from changes to passenger allowances post‐Brexit, although trends arising from COVID‐19 effects are likely to dominate this.

Cross‐border purchasing within GB was more prevalent among drinkers living in Wales and Scotland. These countries have both introduced MUP but in the absence of pre‐implementation data we cannot assess whether the prevalence of cross‐border purchasing rose following the introduction of the policy. Moreover, as a greater proportion of the population of Wales live near the border than is the case for England and Scotland [41], we would expect (all else being equal) to observe higher rates of cross‐border purchasing within GB among people living in Wales. Nonetheless, our findings are consistent with people buying alcohol across the border in England, where there is no MUP, to avoid paying higher prices and suggest a need for further investigation of this topic [17, 18]. We observed significantly higher rates of within‐GB cross‐border purchasing among people living in Wales than those living in Scotland, which might be because people in Wales having to travel less far, on average, than those in Scotland to reach the English border. These data suggest reducing the availability of cheaper alcohol in England may enhance the impact of MUP strategies in Wales and Scotland [11, 17, 18].

Cross‐border purchasing was also reported by a higher proportion of drinkers from more advantaged compared with less advantaged social grades. Differences between social grades were more pronounced for cross‐border purchasing abroad, consistent with advantaged groups being more likely to more frequently travel overseas than those with lower incomes [42], providing greater opportunity to purchase cheaper alcohol abroad. Cross‐border purchasing within GB was also marginally higher among more advantaged drinkers. The extent to which this was intentional (i.e., travelling to other countries within GB for the express purpose of buying alcohol) or circumstantial (i.e., buying alcohol while travelling to other countries within GB for other reasons) is unclear. In any case, this result appears to be inconsistent with MUP as a driver for cross‐border purchasing within GB, as we would expect people with lower incomes to be most affected by MUP. However, the impact of MUP on purchasing behaviour will depend not only on a person's exposure (whether they buy cheap alcohol) and susceptibility (whether they can afford to pay higher prices or have to cut down), but also their ability to mitigate (whether they are able to travel—practically and financially).

Illicit and home‐brewed alcohol purchasing remained relatively rare (<5% of this heavier drinking subsample of the population) over the study period, with no evidence of an increase over time. This indicates that heavier drinkers are not increasing their use of these strategies to reduce their expenditure on alcohol during the cost‐of‐living crisis. The proportion of drinkers reporting having purchased illicit alcohol fell considerably in Scotland from 5.7% to 2.4%, but did not change substantially in England or Wales. This may reflect particular features of heavy drinking in Scotland (e.g., high levels of consuming spirits, which are often part of illicit production) or simply transient trends in illicit markets. As above, the introduction of MUP may have played a role in curtailing illicit alcohol purchasing but there is no obvious mechanism for this, our data cannot provide robust evidence of a causal relationship and previous evidence provides little indication of changes in illicit purchasing [43].

To our knowledge, this is the first study to provide individual‐level evidence on cross‐border alcohol purchasing in GB. Strengths of this study include the large, representative sample and the repeat cross‐sectional design. There were also limitations. Alcohol purchasing data were self‐reported and related to past‐6‐month purchases, introducing scope for reporting and recall bias. The 6‐month coverage period for alcohol purchases should not affect the shape of trends over time (because it affected each monthly wave in the same way) but estimates of prevalence may be lagged as a result. Participants were not asked about the frequency or quantity of cross‐border, illicit or home‐brewed alcohol purchasing so we were not able to distinguish between occasional and regular use of these price‐minimising strategies. The assessment of home brewing was in the context of source of alcohol purchasing, which may have led us to underestimate its prevalence if people interpreted it as only being relevant if they had bought alcohol brewed by someone else. The measure of socioeconomic position was imperfect, as the C2DE group includes varied occupations and some ‘manual’ workers are likely to be on high incomes. In addition, macroeconomic changes during the COVID‐19 pandemic may have had an impact on social grade (e.g., as a result of rising unemployment or disproportionate impacts of the pandemic on different economic sectors [44]). No data were available on alcohol purchasing prior to the implementation of MUP in Scotland or Wales, so we cannot be sure that any between‐country differences were driven by MUP rather than other factors. In addition, our time series began during the COVID‐19 pandemic, which is likely to have affected our baseline and makes interpretation of trends difficult. Without pre‐pandemic data, we cannot conclusively determine the extent to which changes in cross‐border alcohol purchases were influenced by the easing of pandemic restrictions. Finally, our models did not account for seasonal variation in alcohol purchasing. Visual inspection of the data (Figure 1) did not suggest a clear seasonal pattern, and although estimates of changes in prevalence from the start to end of this period were collected in different calendar months, an extended analysis using data up to October 2023 produced a very similar pattern of results.

In conclusion, the proportion of increasing‐ and higher‐risk drinkers in GB reporting cross‐border alcohol purchases increased significantly between October 2020 and August 2023, due to an increase in people buying alcohol abroad as international travel resumed post‐COVID. Cross‐border alcohol purchases within GB were more commonly reported by drinkers in Wales and Scotland. The proportion reporting illicit alcohol purchases was rare and did not change substantially in England or Wales over this period, but fell by half in Scotland. Home‐brewed alcohol purchases remained rare and did not change significantly over time.

## AUTHOR CONTRIBUTIONS

SEJ, MO, CA, JH and JB conceived and designed the study. SEJ analysed the data and drafted the manuscript. MO, CA, JH and JB revised the manuscript critically for important intellectual content. All authors approved the final version.

## FUNDING INFORMATION

Cancer Research UK (PRCRPG‐Nov21\100002) funded the Smoking Toolkit Study data collection and Sarah E. Jackson's salary.

## CONFLICT OF INTEREST STATEMENT

Jamie Brown has received unrestricted research funding from Pfizer and J&J, who manufacture smoking cessation medications. All authors declare no financial links with alcohol companies or their representatives.

## ETHICS STATEMENT

Ethical approval for the STS was granted originally by the UCL Ethics Committee (ID 0498/001). The data are not collected by UCL and are anonymized when received by UCL.

## Supporting information


**Data S1:** Supporting Information.

## Endnote

^1^
Note these two figures do not sum to the cross‐border total because the responses are not mutually exclusive and some people reported doing both.
