# Supplementary material for: Trends in purchasing cross‐border, illicit and home‐brewed alcohol: A population study in Great Britain, 2020–2023
Source: Drug Alcohol Rev. 2024 Mar 21;43(5):1160–71. doi: 10.1111/dar.13838 (PMC11753483; doi:10.1111/dar.13838)
Supplement: Supplementary file 1 — Data S1: Supporting Information. [file DAR-43-1160-s001.docx]

##### Table S1. Comparison of all participants surveyed in eligible waves with the analysed sample of increasing- and higher-risk drinkers

|  | **All participants in eligible waves**  **(*n*^1^=79,352)** | **Analysed sample of increasing- and higher-risk drinkers**  **(*n*^1^=22,086)** |
| --- | --- | --- |
| Country of residence |  |  |
| England | 82.9% | 86.4% |
| Wales | 5.5% | 4.9% |
| Scotland | 11.6% | 8.7% |
| Age, years |  |  |
| Mean (SD) | 46.1 (16.8) | 48.7 (18.5) |
| 16-24 | 12.8% | 11.5% |
| 25-34 | 18.0% | 17.2% |
| 35-44 | 17.1% | 15.9% |
| 45-54 | 19.5% | 16.7% |
| 55-64 | 16.6% | 15.4% |
| ≥65 | 16.0% | 23.3% |
| Gender |  |  |
| Men | 61.3% | 48.6% |
| Women | 38.0% | 50.7% |
| Other | 0.8% | 0.6% |
| Missing, *n***^1^** | 26 | 163 |
| Occupational social grade |  |  |
| ABC1 (more advantaged) | 61.5% | 65.9% |
| C2DE (less advantaged) | 38.5% | 44.1% |

^1^ Unweighted sample size.

Note: Data are shown as weighted column percentages, unless otherwise specified. There were some missing data on gender (unweighted *n*s indicated in the table); valid percentages are shown for ease of interpretation.

##### Table S2. Changes in purchasing cross-border, illicit and home-brewed alcohol among increasing- and higher-risk drinkers in Great Britain between October 2020 and October 2023

|  | **Cross-border alcohol** | | | **Cross-border alcohol within GB** | | | **Cross-border alcohol abroad** | | |
| --- | --- | --- | --- | --- | --- | --- | --- | --- | --- |
|  | % [95% CI] | | Prevalence ratio Oct 20 – Oct 23 [95% CI] | % [95% CI] | | Prevalence ratio Oct 20 – Oct 23 [95% CI] | % [95% CI] | | Prevalence ratio Oct 20 – Oct 23 [95% CI] |
|  | October 2020^1^ | October 2023^1^ |  | October 2020^1^ | October 2023^1^ |  | October 2020^1^ | October 2023^1^ |  |
| All increasing- and higher-risk drinkers | 8.1 [6.9-9.5] | 13.1 [11.4-14.9] | 1.61 [1.32-1.98] | 3.4 [2.6-4.3] | 5.5 [4.4-6.7] | 1.63 [1.20-2.21] | 5.1 [4.1-6.3] | 8.3 [7.0-9.9] | 1.64 [1.25-2.14] |
|  |  |  |  |  |  |  |  |  |  |
| Country |  |  |  |  |  |  |  |  |  |
| England | 7.6 [6.3-9.2] | 12.2 [10.3-14.3] | 1.61 [1.29-2.07] | 2.6 [1.9-3.6] | 4.5 [3.4-5.9] | 1.70 [1.11-2.47] | 5.2 [4.1-6.6] | 8.3 [6.8-10.2] | 1.60 [1.30-2.34] |
| Wales | 17.4 [12.3-24.1] | 18.3 [13.8-24.0] | 1.05 [1.29-2.00] | 14.8 [10.2-21.0] | 12.9 [9.0-18.1] | 0.87 [2.30-4.35] | 3.9 [1.6-9.4] | 7.3 [4.6-11.4] | 1.86 [0.77-1.45] |
| Scotland | 8.3 [5.9-11.6] | 16.4 [13.1-20.4] | 1.98 [0.67-1.02] | 4.5 [2.9-6.9] | 8.4 [5.9-11.7] | 1.87 [0.41-0.75] | 4.5 [2.7-7.5] | 9.4 [7.0-12.6] | 2.09 [0.89-1.67] |
|  |  |  |  |  |  |  |  |  |  |
| Social grade |  |  |  |  |  |  |  |  |  |
| ABC1 (more advantaged) | 9.1 [7.6-10.9] | 15.8 [13.7-18.1] | 1.73 [1.36-2.14] | 3.7 [2.8-4.8] | 6.7 [5.3-8.4] | 1.83 [1.22-2.37] | 6.1 [4.7-7.7] | 10.1 [8.4-12.1] | 1.66 [1.32-2.38] |
| C2DE (less advantaged) | 6.6 [4.8-9.0] | 8.6 [6.3-11.7] | 1.31 [0.50-0.78] | 2.9 [1.8-4.5] | 3.5 [2.2-5.4] | 1.21 [0.42-0.82] | 3.6 [2.3-5.6] | 5.3 [3.5-8.0] | 1.48 [0.48-0.84] |
|  | **Illicit alcohol** | | | **Home-brewed alcohol** | | |  |  |  |
|  | % [95% CI] | | Prevalence ratio Oct 20 – Oct 23 [95% CI] | % [95% CI] | | Prevalence ratio Oct 20 – Oct 23 [95% CI] |  |  |  |
|  | October 2020^1^ | October 2023^1^ |  | October 2020^1^ | October 2023^1^ |  |  |  |  |
| All increasing- and higher-risk drinkers | 4.6 [3.7-5.7] | 4.4 [3.5-5.6] | 0.97 [0.73-1.29] | 3.3 [2.6-4.3] | 3.2 [2.4-4.2] | 0.96 [0.67-1.37] |  |  |  |
|  |  |  |  |  |  |  |  |  |  |
| Country |  |  |  |  |  |  |  |  |  |
| England | 4.5 [3.5-5.8] | 4.7 [3.6-6.1] | 1.05 [0.73-1.44] | 3.3 [2.5-4.4] | 3.3 [2.4-4.6] | 1.01 [0.65-1.38] |  |  |  |
| Wales | 3.3 [1.5-6.9] | 3.0 [1.2-6.9] | 0.91 [0.34-1.13] | 3.3 [1.6-6.5] | 3.8 [1.8-8.0] | 1.17 [0.56-1.64] |  |  |  |
| Scotland | 5.6 [3.7-8.4] | 2.3 [1.3-4.1] | 0.41 [0.56-2.29] | 3.2 [1.9-5.6] | 1.8 [0.8-3.8] | 0.55 [0.38-1.36] |  |  |  |
|  |  |  |  |  |  |  |  |  |  |
| Social grade |  |  |  |  |  |  |  |  |  |
| ABC1 (more advantaged) | 4.0 [3.0-5.2] | 4.6 [3.5-6.0] | 1.16 [0.79-1.66] | 3.4 [2.6-4.6] | 3.4 [2.5-4.7] | 0.99 [0.65-1.41] |  |  |  |
| C2DE (less advantaged) | 5.5 [3.8-7.7] | 4.1 [2.7-6.4] | 0.76 [0.64-1.37] | 3.2 [2.0-5.1] | 2.8. [1.5-5.0] | 0.88 [0.42-1.12] |  |  |  |

CI, confidence interval.

^1^ Weighted prevalence from logistic regression models on all increasing- and higher-risk drinkers in Great Britain and (for estimates by region and social grade) allowing an interaction between survey wave and the moderator of interest, modelled non-linearly using restricted cubic splines.


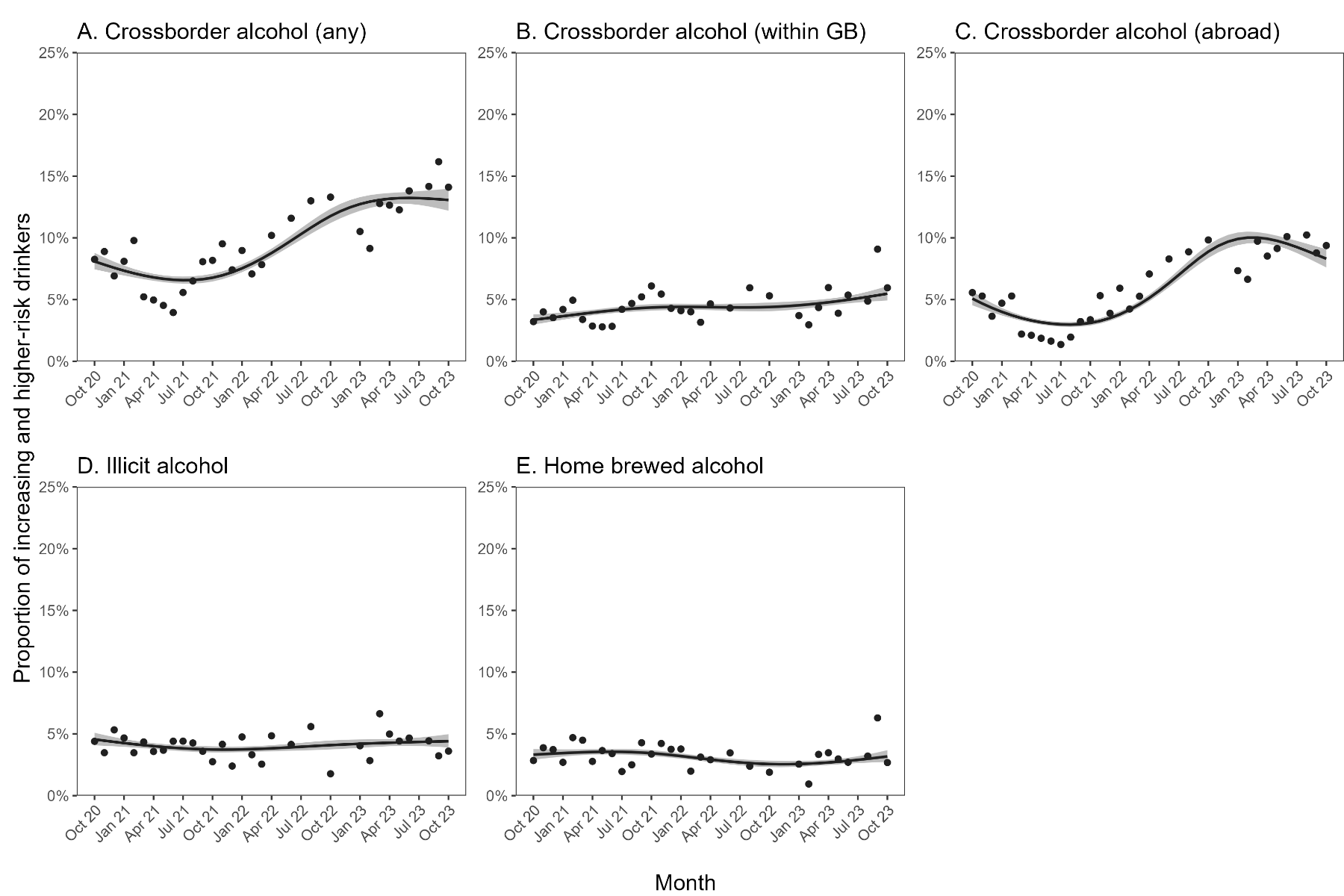


##### Figure S1. Trends in the proportion of increasing- and higher-risk drinkers in Great Britain (*n*=21,663) who reported purchasing cross-border, illicit, and home-brewed alcohol, October 2020 to October 2023. Lines represent modelled weighted prevalence by monthly survey wave, modelled non-linearly using restricted cubic splines (four knots). Shaded bands represent standard errors. Points represent observed weighted prevalence by month.


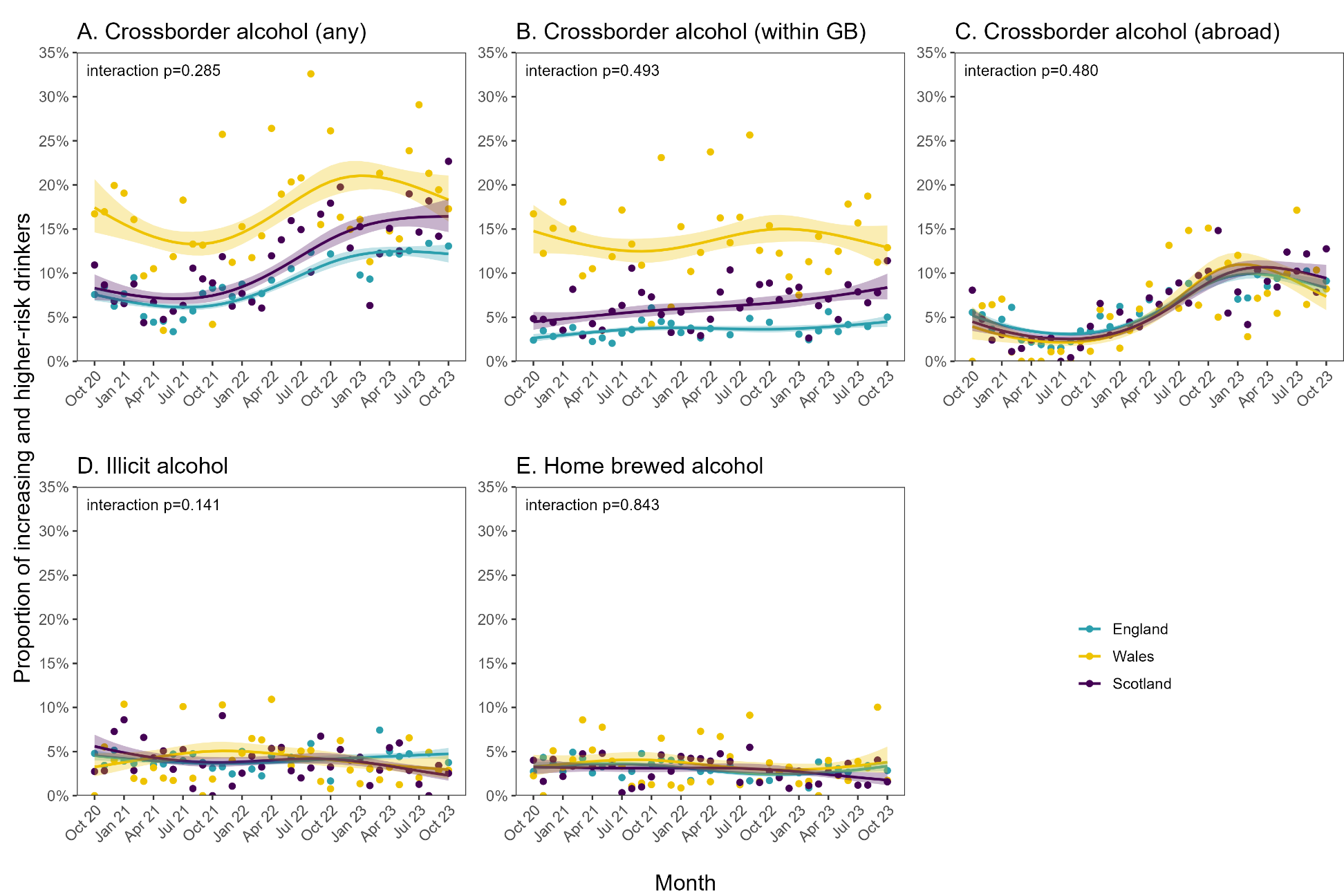


##### Figure S2. Trends in the proportion of increasing- and higher-risk drinkers in England (*n*=15,060), Wales (*n*=2,544), and Scotland (*n*=5,412) who reported purchasing cross-border, illicit, and home-brewed alcohol, October 2020 to October 2023. Lines represent modelled weighted prevalence by monthly survey wave, modelled non-linearly using restricted cubic splines (four knots). Shaded bands represent standard errors. Points represent observed weighted prevalence by month.


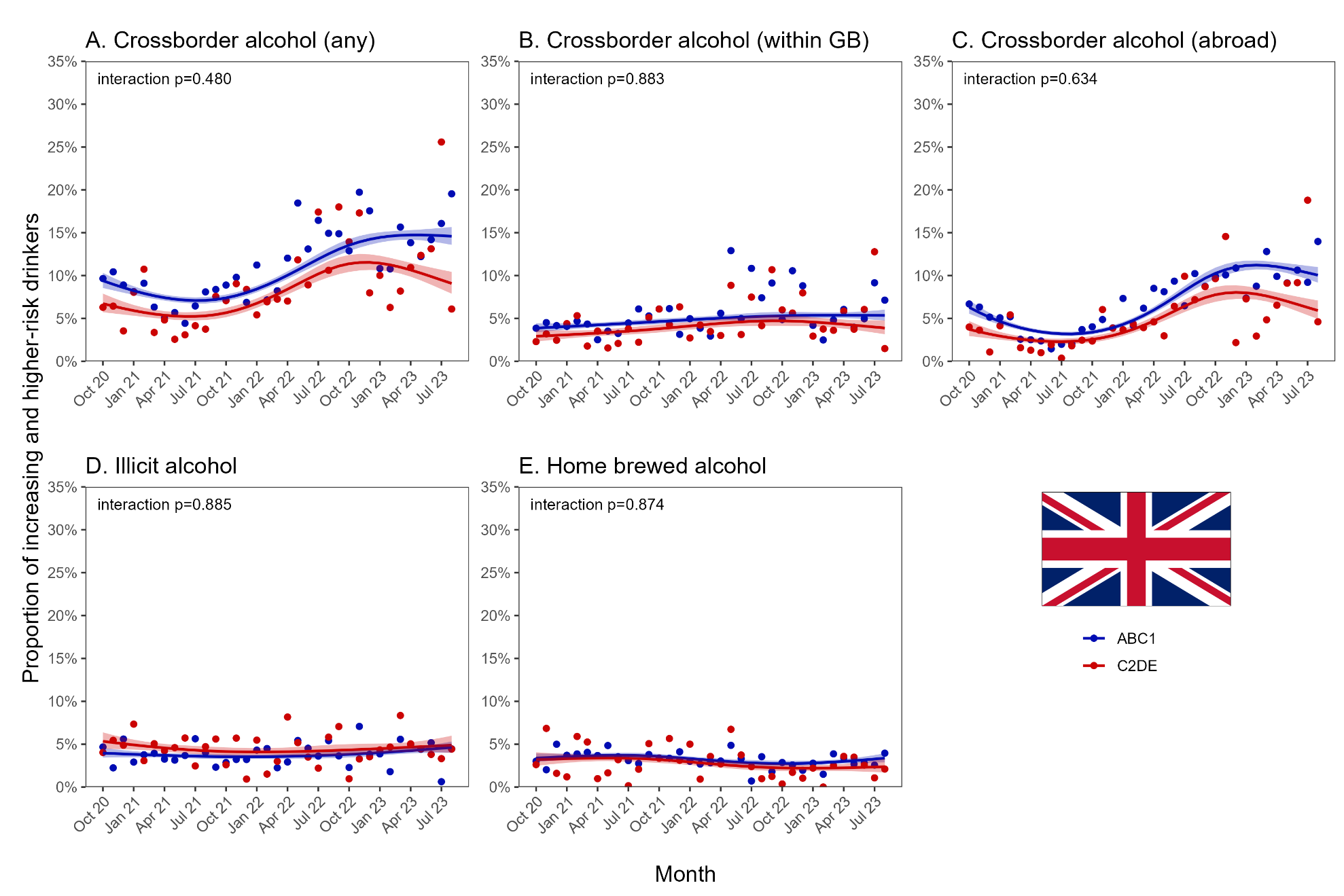


##### Figure S3. Social grade-specific trends in cross-border, illicit, and home-brewed alcohol purchasing among increasing and higher-risk drinkers in Great Britain, October 2020 to August 2023. Lines represent modelled weighted prevalence by monthly survey wave, modelled non-linearly using restricted cubic splines (four knots). Shaded bands represent standard errors. Points represent observed weighted prevalence by month.


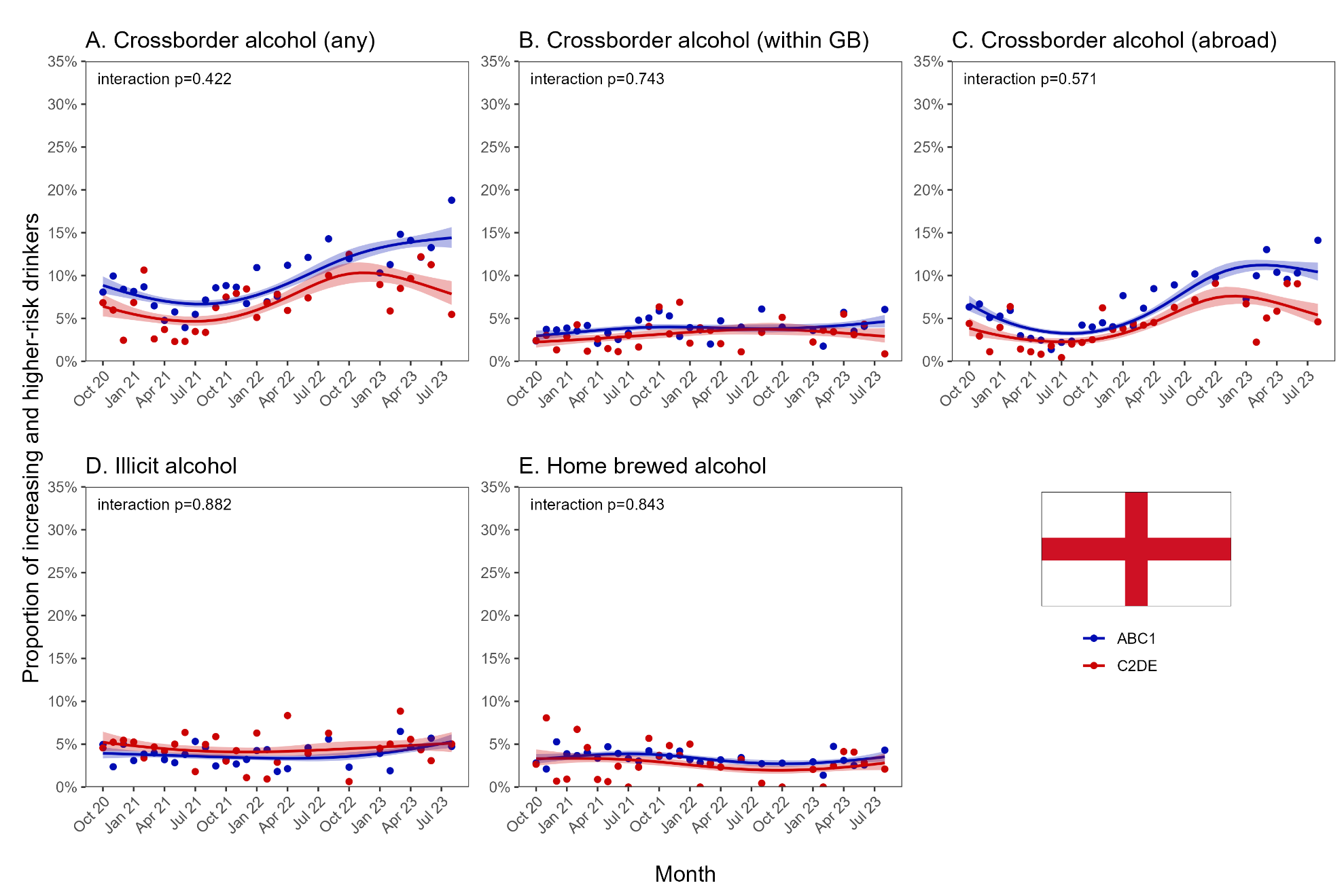


##### Figure S4. Social grade-specific trends in cross-border, illicit, and home-brewed alcohol purchasing among increasing and higher-risk drinkers in England, October 2020 to August 2023. Lines represent modelled weighted prevalence by monthly survey wave, modelled non-linearly using restricted cubic splines (four knots). Shaded bands represent standard errors. Points represent observed weighted prevalence by month.


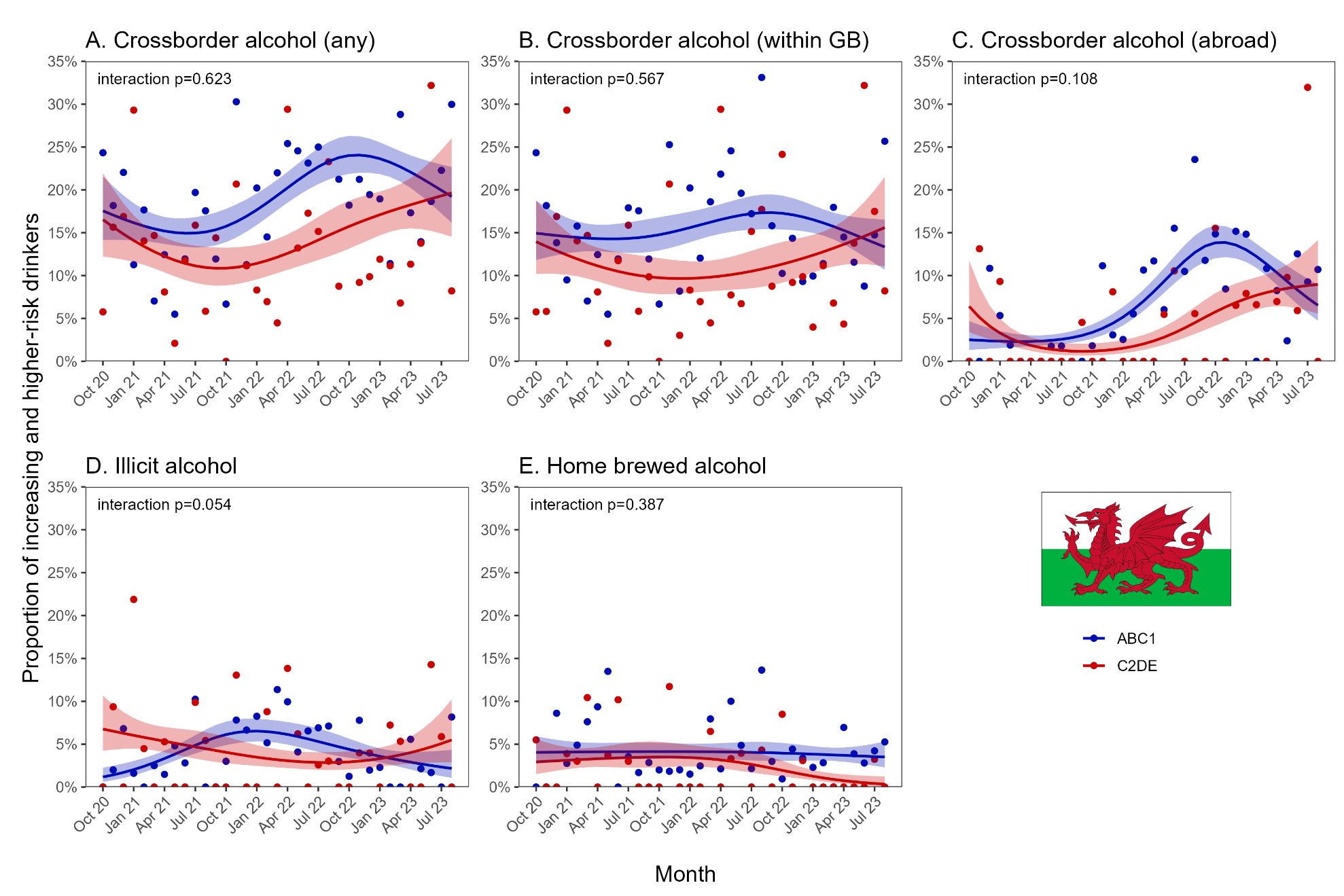


##### Figure S5. Social grade-specific trends in cross-border, illicit, and home-brewed alcohol purchasing among increasing and higher-risk drinkers in Wales, October 2020 to August 2023. Lines represent modelled weighted prevalence by monthly survey wave, modelled non-linearly using restricted cubic splines (four knots). Shaded bands represent standard errors. Points represent observed weighted prevalence by month.


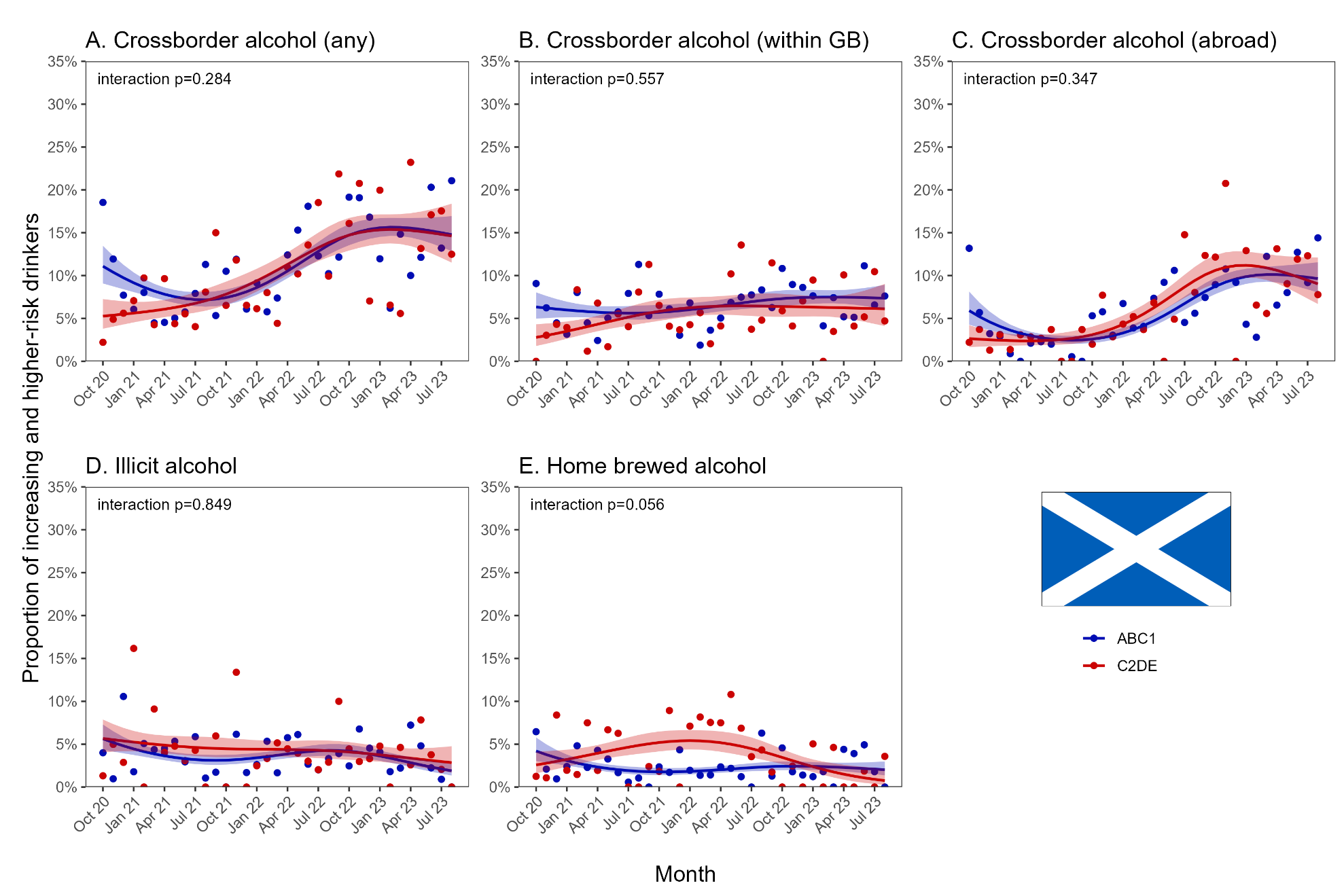


##### Figure S6. Social grade-specific trends in cross-border, illicit, and home-brewed alcohol purchasing among increasing and higher-risk drinkers in Scotland, October 2020 to August 2023. Lines represent modelled weighted prevalence by monthly survey wave, modelled non-linearly using restricted cubic splines (four knots). Shaded bands represent standard errors. Points represent observed weighted prevalence by month.

##### Table S3. Social grade-specific changes in cross-border, illicit, and home-brewed alcohol purchasing among increasing- and higher-risk drinkers in England, Wales, and Scotland between October 2020 and August 2023

|  | **Cross-border alcohol** | | |  | **Cross-border alcohol within GB** | | |  | **Cross-border alcohol abroad** | | |
| --- | --- | --- | --- | --- | --- | --- | --- | --- | --- | --- | --- |
|  | % [95% CI] | | Prevalence ratio Oct 20 – Aug 23 [95% CI] |  | % [95% CI] | | Prevalence ratio Oct 20 – Aug 23 [95% CI] |  | % [95% CI] | | Prevalence ratio Oct 20 – Aug 23 [95% CI] |
|  | October 2020^1^ | August 2023^1^ |  |  | October 2020^1^ | August 2023^1^ |  |  | October 2020^1^ | August 2023^1^ |  |
|  |  |  |  |  |  |  |  |  |  |  |  |
| England |  |  |  |  |  |  |  |  |  |  |  |
| ABC1 (more advantaged) | 8.9 [7.1-11.0] | 14.4 [12.2-16.9] | 1.63 [1.23-2.21] |  | 3.0 [2.0-4.3] | 4.6 [3.4-6.3] | 1.56 [0.93-2.66] |  | 6.7 [5.1-8.7] | 10.4 [8.5-12.7] | 1.55 [1.15-2.25] |
| C2DE (less advantaged) | 6.4 [4.3-9.4] | 7.9 [5.5-11.0] | 1.23 [0.71-2.22] |  | 2.2 [1.2-4.3] | 2.9 [1.7-5.1] | 1.30 [0.50-3.40] |  | 3.9 [2.3-6.4] | 5.4 [3.5-8.2] | 1.40 [0.70-3.04] |
|  |  |  |  |  |  |  |  |  |  |  |  |
| Wales |  |  |  |  |  |  |  |  |  |  |  |
| ABC1 (more advantaged) | 17.6 [11.4-26.0] | 19.2 [13.6-26.4] | 1.09 [0.64-1.99] |  | 14.9 [9.4-23.0] | 13.3 [8.6-20.2] | 0.89 [0.46-1.92] |  | 2.5 [0.7-8.4] | 6.5 [3.5-12.0] | 2.60 [0.75-25.1] |
| C2DE (less advantaged) | 16.5 [9.1-28.2] | 19.6 [10.7-33.3] | 1.19 [0.50-3.03] |  | 14.0 [7.5-24.6] | 15.6 [7.9-28.6] | 1.12 [0.39-2.93] |  | 6.4 [1.8-20.1] | 9.0 [3.5-21.2] | 1.40 [0.28-17.7] |
|  |  |  |  |  |  |  |  |  |  |  |  |
| Scotland |  |  |  |  |  |  |  |  |  |  |  |
| ABC1 (more advantaged) | 11.1 [7.5-16.2] | 14.8 [11.2-19.2] | 1.33 [0.83-2.23] |  | 6.3 [3.9-10.1] | 7.4 [5.0-10.7] | 1.16 [0.60-2.25] |  | 5.9 [3.1-11.0] | 9.7 [6.7-13.7] | 1.63 [0.80-4.16] |
| C2DE (less advantaged) | 5.3 [2.8-9.8] | 14.6 [9.1-22.7] | 2.77 [1.31-6.89] |  | 2.8 [1.2-6.5] | 6.1 [2.8-12.9] | 2.20 [0.60-8.47] |  | 2.6 [1.1-6.5] | 9.0 [5.0-15.8] | 3.41 [1.18-14.4] |
|  |  |  |  |  |  |  |  |  |  |  |  |
|  | **Illicit alcohol** | | |  | **Home-brewed alcohol** | | |  |  |  |  |
|  | % [95% CI] | | Prevalence ratio Oct 20 – Aug 23 [95% CI] |  | % [95% CI] | | Prevalence ratio Oct 20 – Aug 23 [95% CI] |  |  |  |  |
|  | October 2020^1^ | August 2023^1^ |  |  | October 2020^1^ | August 2023^1^ |  |  |  |  |  |
|  |  |  |  |  |  |  |  |  |  |  |  |
| England |  |  |  |  |  |  |  |  |  |  |  |
| ABC1 (more advantaged) | 3.9 [2.8-5.4] | 5.3 [4.0-7.1] | 1.34 [0.85-2.22] |  | 3.2 [2.3-4.5] | 3.5 [2.5-4.9] | 1.09 [0.65-1.76] |  |  |  |  |
| C2DE (less advantaged) | 5.2 [3.4-7.9] | 5.1 [3.3-7.9] | 0.98 [0.49-2.02] |  | 3.3 [1.9-5.7] | 2.8 [1.4-5.5] | 0.85 [0.29-2.19] |  |  |  |  |
|  |  |  |  |  |  |  |  |  |  |  |  |
| Wales |  |  |  |  |  |  |  |  |  |  |  |
| ABC1 (more advantaged) | 1.2 [0.3-4.1] | 2.2 [0.5-8.3] | 1.84 [0.16-19.7] |  | 4.0 [1.9-8.5] | 3.5 [1.5-7.9] | 0.87 [0.23-2.88] |  |  |  |  |
| C2DE (less advantaged) | 6.8 [2.7-16.1] | 5.5 [1.5-17.8] | 0.81 [0.06-4.41] |  | 2.9 [0.8-10.2] | 0.3 [0.0-4.2] | 0.12 [0.00-1.96] |  |  |  |  |
|  |  |  |  |  |  |  |  |  |  |  |  |
| Scotland |  |  |  |  |  |  |  |  |  |  |  |
| ABC1 (more advantaged) | 5.6 [3.3-9.3] | 1.9 [1.0-3.7] | 0.34 [0.13-0.78] |  | 4.2 [2.2-7.8] | 2.0 [0.9-4.3] | 0.48 [0.15-1.36] |  |  |  |  |
| C2DE (less advantaged) | 5.7 [2.9-10.7] | 2.8 [1.0-7.7] | 0.50 [0.10-1.62] |  | 2.6 [1.0-6.4] | 0.8 [0.1-5.7] | 0.29 [0.00-2.16] |  |  |  |  |
|  |  |  |  |  |  |  |  |  |  |  |  |

CI, confidence interval.

^1^ Weighted prevalence from logistic regression models on all increasing- and higher-risk drinkers in a given country, allowing an interaction between survey wave and social grade, modelled non-linearly using restricted cubic splines.
